# Asymptomatic* Leishmania* Infected Children: A Seroprevalence and Molecular Survey in a Rural Area of Fars Province, Southern Iran

**DOI:** 10.1155/2018/8167247

**Published:** 2018-05-15

**Authors:** Akram Layegh Gigloo, Bahador Sarkari, Zahra Rezaei, Gholam Reza Hatam, Mohammad Hassan Davami

**Affiliations:** ^1^Department of Parasitology and Mycology, School of Medicine, Shiraz University of Medical Sciences, Shiraz, Iran; ^2^Basic Sciences in Infectious Diseases Research Center, Shiraz University of Medical Sciences, Shiraz, Iran; ^3^Professor Alborzi Clinical Microbiology Research Center, Nemazee Hospital, Shiraz University of Medical Sciences, Shiraz, Iran; ^4^Department of Parasitology and Mycology, School of Medicine, Jahrom University of Medical Sciences, Jahrom, Iran

## Abstract

The current study aimed to evaluate the seroprevalence of visceral leishmaniasis in asymptomatic healthy children in a rural area of Fars province, Southern Iran. Blood samples were taken from 617 asymptomatic healthy children and serum samples along with buffy coat were separated from the blood. The serum samples were assessed for antibodies against* Leishmania infantum* by an indirect ELISA and the buffy coats were tested for the presence of* L. infantum* DNA by molecular method. Of the 617 recruited children, 297 (48.1%) were female and 317 (51.4%) were male. Anti-*Leishmania* antibodies were detected in 17 (2.8%) of the children. From those 17 seropositive cases, 5 (29.4%) were male and 12 (70.6%) cases were female. Children aged 5–8 years had the highest seroprevalence rate; however, no associations were found between seropositivity to* Leishmania* and gender or age of the children. Moreover,* L. infantum* DNA was detected in buffy coat of 8 (1.3%) of 617 children. Three of the PCR-positive cases were seropositive whereas 14 of seropositive subjects (82.3%) were PCR-negative. Findings of the current study revealed a considerable subclinical leishmanial infection in children in the studied rural area in the south of Iran. Results of the current study could be used for surveillance, prevention, and control of VL in the area.

## 1. Introduction

Visceral leishmaniasis (VL), commonly known as kala-azar, is an important public health problem in several countries of the world, including Iran [1–5].* Leishmania infantum* is mainly responsible for VL while visceral infection due to* L. tropica* has also been reported [6]. Although VL has been reported from all provinces of Iran, the main foci of VL are northwest and southern parts of the country [2, 3, 5, 7]. In the southern part, VL is continuously reported from Nurabad, Darab, Kazerun, and Firuzabad townships and the disease is much more prevalent in tribal areas of the province [7, 8]. During 1999–2014 in Fars province, southwestern Iran, 380 VL cases have been recorded, giving an average annual hospital admission of 23.75 cases [5]. Dogs are the main reservoir host of VL in Iran, although feline leishmaniasis has also been reported and cats may have a role in the epidemiology of VL [9, 10].

Seroprevalence of VL varies from less than 1% to more than 25% in different areas of Iran [11–15]. A seroepidemiological study for VL among healthy blood donors in the south of Iran showed a seroprevalence rate of 1.4% among 2003 healthy blood donors [14].

In 2005, Alborzi et al. evaluated the asymptomatic VL infection in 388 healthy individuals living in two endemic foci of VL (Ghir Karzin and Sar Mashhad) in the south of Iran. They found* Leishmania* DNA in 24.5% of the subjects while 54.6% of the examined subjects were seropositive by IFAT. While most (45%) of seropositive cases were PCR-positive, in 55% of seropositive cases DNA was not detected [16]. A study in Dehloran, west of Iran, found that 2.3% of children up to 12 years are seropositive for VL [12].

In another study in Kerman province, southeastern Iran, seropositivity of VL was assessed on 862 children up to 12 years from nomadic tribes. Results of the study showed that 2.6% of children are seropositive for VL. Children aged 5–8 years had the highest seroprevalence rate [17]. In Fakhar et al. study in Qom province, central Iran, 1.7% of healthy subjects were seropositive for VL [18]. Asgari et al. evaluated the seroprevalence of VL in children in Qashqai tribes in Fars province, Southern Iran, and found that 1.86% of children aged up to 12 years are seropositive for VL [8].

The objective of the current study was to determine the seroprevalence of VL in asymptomatic children in a rural area in Fars province, Southern Iran. The study also aimed to find out the* Leishmania* infection, by the molecular method, in the asymptomatic individuals. Moreover, the study aimed to pinpoint the possible contributory factors in seroprevalence of VL in the area.

## 2. Materials and Methods

### 2.1. Study Area

This seroepidemiological and molecular study of VL was performed in Sar Mashhad region at a 29.294N latitude and 51.701E longitude, located in Kazeroun Township in Fars province, Southern Iran. At the 2006 census, its population was 5,000, in 1000 families. The area has a moderate winter and hot summer. The district is situated near the border of Bushehr province. Sar Mashhad is an area where VL has been continuously reported during the last decades [5, 7].

### 2.2. Blood Sampling

Considering the population of children in the area, sampling was done in three villages in the area in May 2017. Fresh blood samples (5 mL) were obtained from 617 asymptomatic healthy children and serum along with buffy coat was separated from the blood and stored at −20°C until use. All serum samples were assessed for antibodies against* L. infantum* by an indirect ELISA. Moreover, the buffy coat of each subject was tested for the presence of* L. infantum* DNA by molecular (PCR) method.

The study protocol was approved by the ethical review committee of the Shiraz University of Medical Sciences (SUMS). Informed consent was given by children guardians. Subjects with positive results were called to consult with their physician for any sign or symptoms of the disease.

### 2.3. Antigen Preparation for ELISA

*L. infantum* crude soluble antigen was prepared from a reference human strain.* Leishmania* promastigote was mass cultivated in an RPMI 1640 medium containing 10% inactivated FBS (fetal bovine serum) at 26°C. The promastigote pellet was collected by centrifugation, washed three times with PBS, and lysed by five cycles of freezing and thawing followed by sonication. The protein content was estimated by Bradford method and the extracted antigen was stored at −20°C until use.

### 2.4. ELISA for Detection of Anti-*Leishmania* Antibodies

ELISA was performed to detect anti-*Leishmania* antibodies in sera samples [19]. The flat-bottom 96-well microplates (Corning, USA) were coated with 100 *μ*L/well of purified antigen at a concentration of 5 *μ*g/mL in 0.1 M carbonate/bicarbonate (pH 9.6) buffer by overnight incubation at 4°C. Unbound antigens were removed by washing the plate five times in phosphate buffered saline-Tween 20 (PBST, pH 7.4 containing 0.05% Tween 20). Blocking was performed with 200 *μ*L of 3% nonfat skimmed milk in PBST for 2 hours at room temperature. Then, the wells were washed, 5 times with washing buffer. Diluted serum samples (1 : 100 in PBST) were applied to the plates and incubated for 1 hour at room temperature. The plates were washed as before and 100 *μ*L of 1 : 4000 dilution of horseradish peroxidase-conjugated goat anti-human IgG (Sigma, USA) was added to the plates and incubated for 1.5 hours at room temperature. The plates were then washed as before and incubated with substrate (100 *μ*L/well of 0.4 mg/mL OPD, 0.3% H_2_O_2_ in 0.1 M citrate buffer, pH 5) for 20 minutes. The reaction was stopped by using 1 N H_2_SO_4_. The absorbance at 490 nm was checked with a microplate reader (Bio-Tek, ELx800). Positive sera from VL-confirmed cases were applied in each plate. The cutoff point was fixed at 2SD above the mean of control samples.

### 2.5. DNA Extraction and Molecular Method

For molecular evaluation, DNA was extracted from the buffy coat of the samples, using proteinase K and lysis buffer followed by phenol/chloroform/isoamyl extraction [14]. Extracted DNA was precipitated by using absolute ethanol and was resuspended in 100 *μ*L of double distilled water and stored at 4°C until use. Polymerase chain reaction (PCR) was performed targeting ITS-2 gene of* Leishmania. *A 200 bp fragment of the ITS-2 gene was amplified, using the external primers 5′-AAACTCCTC TCTGGTGCTTGC-3′ (forward) and 5′-AAACAAAGGTTGTCGGGGG-3′ (reverse) and the internal transcribed spacer- (ITS-) 2 gene of* Leishmania* spp. primers: 5′-AAT TCA ACT TCG CGT TGG CC-3′ (forward) and 5′-CCTCTCTTTTTTCTCTGTGC-3′ (reverse). For PCR amplification, the final volume of 25 *μ*L reaction, containing 2 *μ*L of extracted DNA, 1 *μ*L (10 pm) of each primer, 12.5 *μ*L of 1x Taq DNA Polymerase Master Mix RED, and 9.5 *μ*L of DW, was used. The thermal cycling conditions were as follows: initial denaturation at 94.5°C for 5 min, followed by 35 cycles including denaturation at 94°C for 30 s, annealing at 55°C for 30 s, extension at 72°C for 30 s, and a final extension at 72°C for 8 min. PCR products were separated by electrophoresis in 1.5% agarose gel, and stained with gel red.

### 2.6. Analysis of Data

Collected data were analyzed by SPSS software (version 18). Chi-squared and Fisher exact tests were used to determine the association between the seropositivity to VL and sociodemographic features of the subjects. The significance threshold was set at 0.05.

## 3. Results

The study population included 617 healthy children consisting of 319 (51.7%) males and 298 (48.3%) females. The mean age of participants was 9.7 (±10.7) years. Most of the subjects (29.7%) were aged 5–8 years. Subjects were from three main villages, Sar Mashhad (475 cases), Tole Saman (93 cases), and Hossein Abad (49 cases).

Anti-*Leishmania* antibodies were detected in the sera of 17 out of 617 recruited subjects corresponding to seroprevalence of 2.8% in the examined children. From those 17 seropositive cases, 12 cases (70.6%) were female and 5 cases (29.4%) were male. While individuals aged 5–8 years had the highest seropositivity (3.8%) to VL, yet the differences between age groups were not statistically significant (*p* > 0.05).

Furthermore, there were no associations between seropositivity to VL and using the bed net, the presence of dogs in the household, history of kala-azar (based on the claims of the subjects), and residence of the participants.  Table 1 shows the sociodemographic features of children and relative seropositivity to* L. infantum* in this study.

**Figure 1:**
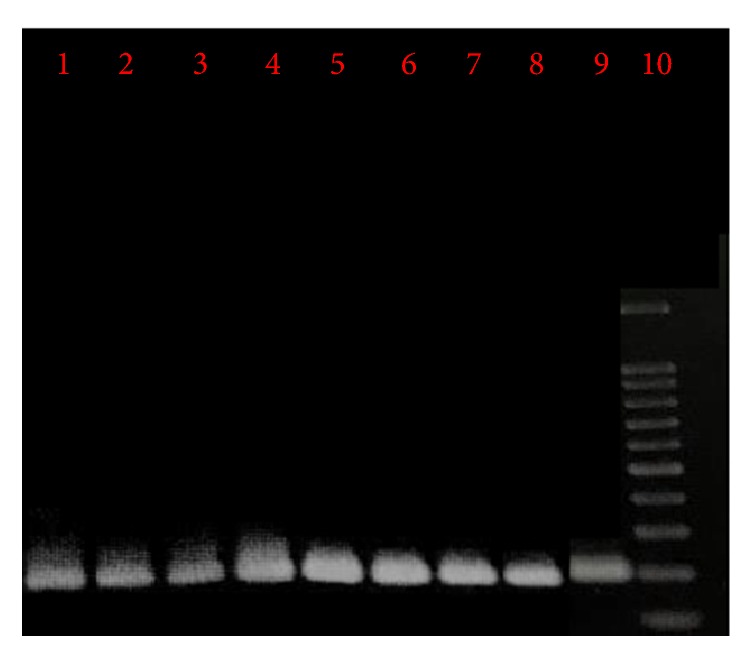
Agarose gel electrophoresis of PCR product of ITS-2 gene of* L. infantum* from buffy coats of healthy children. Lanes 1 to 8: samples isolated from the buffy coat of children. Lane 9: positive control* (L. infantum)*. Lane 10: molecular marker.

PCR detected* L. infantum* DNA in buffy coat of 8 (1.3%) of children.  Figure 1 shows the PCR product of* L. infantum* gene in buffy coats of healthy children. Five of the PCR-positive cases, (62.5%) were female and 3 (37.6%) were male. Association between gender and PCR-positivity was not significant (*p* > 0.05). Three of the PCR-positive cases were seropositive for VL, whereas 14 (82.3%) of seropositive subjects were PCR-negative. A significant association was found between the PCR and seropositivity to* L. infantum *(*p* < 0.05).

## 4. Discussion

The presence of clinical cases of VL cannot be the only indicator for the VL transmission in a given area. Evaluation of the rate of asymptomatic infection among the population of a VL-endemic area seems to be a valuable alternative approach for understanding the VL transmission and also for the implementation of control measurements. For proper maneuver of any prevention and sustainable control and also surveillance program of VL, obtaining data about the epidemiology of VL is necessary. The current study aimed to find out the seroprevalence of VL in asymptomatic healthy children in a rural district in the south of Iran.

In many of VL-endemic areas of Iran and also other VL-endemic areas of the world,* L. infantum* infection remains asymptomatic in certain subjects [14, 16, 20–24]. Therefore,* L. infantum* infection in children living in these areas does not necessarily mean the VL disease. The ratio of asymptomatic cases to clinical cases of VL is reported to be 5.6 : 1 in Ethiopia, 2.6 : 1 to 11 : 1 in Sudan, or 4 : 1 in Kenya [25–27].

A study in Basra, southern Iraq, found that 20% of healthy children are positive for anti-*Leishmania* antibodies, tested by DAT [28]. A seroepidemiological survey in Kars province in Turkey found that 7.4% of dogs are infected with* Leishmania* although no seropositive human case was found during the survey [29].

In a seroprevalence study by Sarkari et al., which was carried out in children in Boyer-Ahmad district, southwest of Iran, anti-*Leishmania* antibodies were detected in 3.1% of children [15]. In the current study, 617 serum samples were obtained from asymptomatic healthy children and were tested for anti-*Leishmania* antibodies by ELISA. Findings of this study revealed a seroprevalence rate of 2.8% for VL in healthy children. The seroprevalence rate seems to be low in comparison with the rate reported from some of the VL-endemic areas of Iran but seems to be considerable in comparison with some other areas [11–15].

In the current study, no association was found between seropositivity to VL and gender of the participants. The findings are consistent with reports from other areas of the country [13, 15, 17, 30]. Moreover, no association was found between seropositivity to VL and history of VL. In fact the VL history of the subjects was not medically confirmed or validated in this study and the consideration was only based on the claims of the subjects. This can be reflected as a limitation of the current study.

In VL, the majority of cases are asymptomatic and do not progress to overt disease [14, 16, 20, 25]. It is noteworthy that the asymptomatic children who develop secondary immunodeficiency disorders are prone to reactive VL disease [31]. Also, asymptomatic humans may constitute a reservoir for VL. These apparently healthy subjects may contribute a risk of distributing the VL in the area.

In the current study, the presence of dogs in the household was not a risk factor for obtaining VL infection. This is consistent with the notion that presence of dogs in the household does not seem to be a significant determinant of VL infection [20]. Findings of the current study further confirmed this notion.

DAT, IFAT,* Leishmania* Skin Test (LST), and ELISA are the main immunoassays used for seroprevalence surveys in different VL-endemic areas [11–16, 32, 33]. Among them, DAT is the most widely used test, due to its simplicity and its high sensitivity and specificity. In the current study, ELISA was used to find out the rate of seropositivity to VL. This format of ELISA has been evaluated along with DAT in our previous study which had a very good agreement with DAT [33].

In the current study, DNA was detected in buffy coat of 8 (1.3%) of the examined healthy children. Only 3 PCR-positive cases were seropositive while most of the seropositive subjects were PCR-negative. In a previous study, Alborzi et al. reported the presence of* Leishmania* DNA in 24.5% of the healthy children in two VL-endemic areas in the south of Iran [16]. A study on 231 healthy subjects living in a kala-azar endemic focus in Nepal revealed that 12.5% of healthy households are positive for* Leishmania* DNA while 20.8% of the subjects were DAT seropositive [21]. It was noticed that positive agreement between seropositivity and PCR-positivity was poor but negative agreement between two methods was good [21]. In the Mediterranean region, PCR-positivity rates among asymptomatic blood donors in Spain were reported to be 5.9% [24].

In the current study, the majority (5 out of 8 cases) of the PCR-positive individuals were seronegative. The reason for this is not completely understood. This might be linked to the time of infection where the patients have recently acquired the infection and the immune system has not yet been adequately stimulated to produce a detectable level of antibodies. It also may be linked to the sensitivity of the method as PCR is a much more sensitive approach for the diagnosis of VL infection than the serological method, in this case ELISA, and would be able to detect more cases of VL infection than the ELISA.

In fact, most of the PCR-positive cases do not develop active VL disease, although our knowledge about this concept is lacking and long-term follow-up surveys should be undertaken to find out the outcome of asymptomatic carriers of* Leishmania*.

## 5. Conclusion

Taken together, findings of the current study revealed that* L. infantum* is a common infection in asymptomatic healthy children in the studied rural area in the south of Iran. PCR-positive healthy individuals may act as a reservoir for VL, although the epidemiological role of these healthy subjects has not properly been clarified. Results of the current study might be used for surveillance, prevention, and control of VL in the area. Further studies are needed to find out the genotype of the parasite and also the reservoirs of VL in the studied area.

## Figures and Tables

**Table 1:** Sociodemographic characteristics of children and relative seropositivity to *L. infantum* in Sar Mashhad district, Southern Iran.

Characteristics	Frequency (number)	Percent (%)	Positive for anti-*Leishmania* antibodies		*p* value
			Frequency (number)	Percent (%)	
*Gender *					
Male	319	51.7	5	1.6	0.084
Female	298	48.3	12	4.0	

*Age group*					
1–4	180	29.4	5	2.8	0.753
5–8	183	29.9	7	3.8	
9–12	182	29.7	4	2.2	
>12	67	10.9	1	1.5	

*Residence area*					
Sar Mashhad	475	77.0	13	2.7	0.918
Tole Saman	93	15.1	3	3.2	
Hossein Abad	49	7.9	1	2.0	

*Educational level*					
Illiterate	260	42.1	8	3.1	0.933
Elementary	342	55.4	9	2.6	
Secondary level	14	2.3	0	0	
High school	1	0.2	0	0	

*Presence of dogs in the household*					
Yes	383	62.1	10	2.6	0.803
No	234	37.9	7	3.0	

*Using bed net*					
Yes	508	82.3	14	2.8	1.00
No	109	17.7	3	2.7	

^*∗*^History* of Kala-azar*					
Yes	86	13.9	2	2.3	1.00
No	531	86.1	15	2.8	

^*∗*^Based on the claims of the subjects.
